# Extended endoscopic supracerebellar infratentorial (EESI) approach for a complex pineal region tumour—a technical note

**DOI:** 10.1007/s00381-018-3797-7

**Published:** 2018-04-23

**Authors:** Saurabh Sinha, Elizabeth Culpin, John McMullan

**Affiliations:** Department of Paediatric Neurosurgery, Sheffield Children’s Hospital, Sheffield, UK

**Keywords:** Extended endoscopic supracerebellar infratentorial approach, Parinaud syndrome, MRI

## Abstract

Endoscopic-assisted approaches have been shown to be a suitable alternative to the standard microscopic approach to pineal region tumours. With extension laterally into the ventricles, the 0° endoscope and microscope have significant limitations. We describe the extended endoscopic supracerebellar infratentorial (EESI) approach using angled endoscopes for a complex pineal region tumour that extended into the lateral ventricle. A 15-year-old boy presented with headaches and ataxia. MRI revealed a pineal region tumour extending into the lateral ventricle. The patient was positioned in the sitting position. The supracerebellar infratentorial corridor was accessed through a small craniotomy. The tumour was resected completely via the endoscope. Postoperatively, the patient’s symptoms resolved completely. We believe that this case highlights the benefit of using the endoscopic extended supracerebellar infratentorial (EESI) approach to resect pineal region lesions that extend beyond the midline.

## Introduction

The benefit of endoscopic-assisted surgery in a patient with a pineal cyst has previously been documented by Gore et al. [1]. Further case reports have also demonstrated the benefit of such an approach [2–6]. However, in the majority of cases, the lesions were pineal cysts or small lesions located in the midline and could have been accessed with a microscope in a similar fashion. Where the lesions were larger or not cystic, the main surgical aim was to obtain a biopsy and debulk the lesion.

The use of angled endoscopes in extended endoscopic skull base approaches has allowed increasingly complex tumours that extend beyond the midline to be removed via an extended trans-sphenoidal approach.

We have modified these skull base techniques and principles to perform an extended endoscopic supracerebellar infratentorial (EESI) approach in the sitting position to excise a large pineal region tumour that extended laterally into the ventricle.

We believe that this technique may allow more complex lesions within this region to be accessed and removed without the need for cortical resection or more than one approach.

### Clinical presentation

**Fig. 1:**
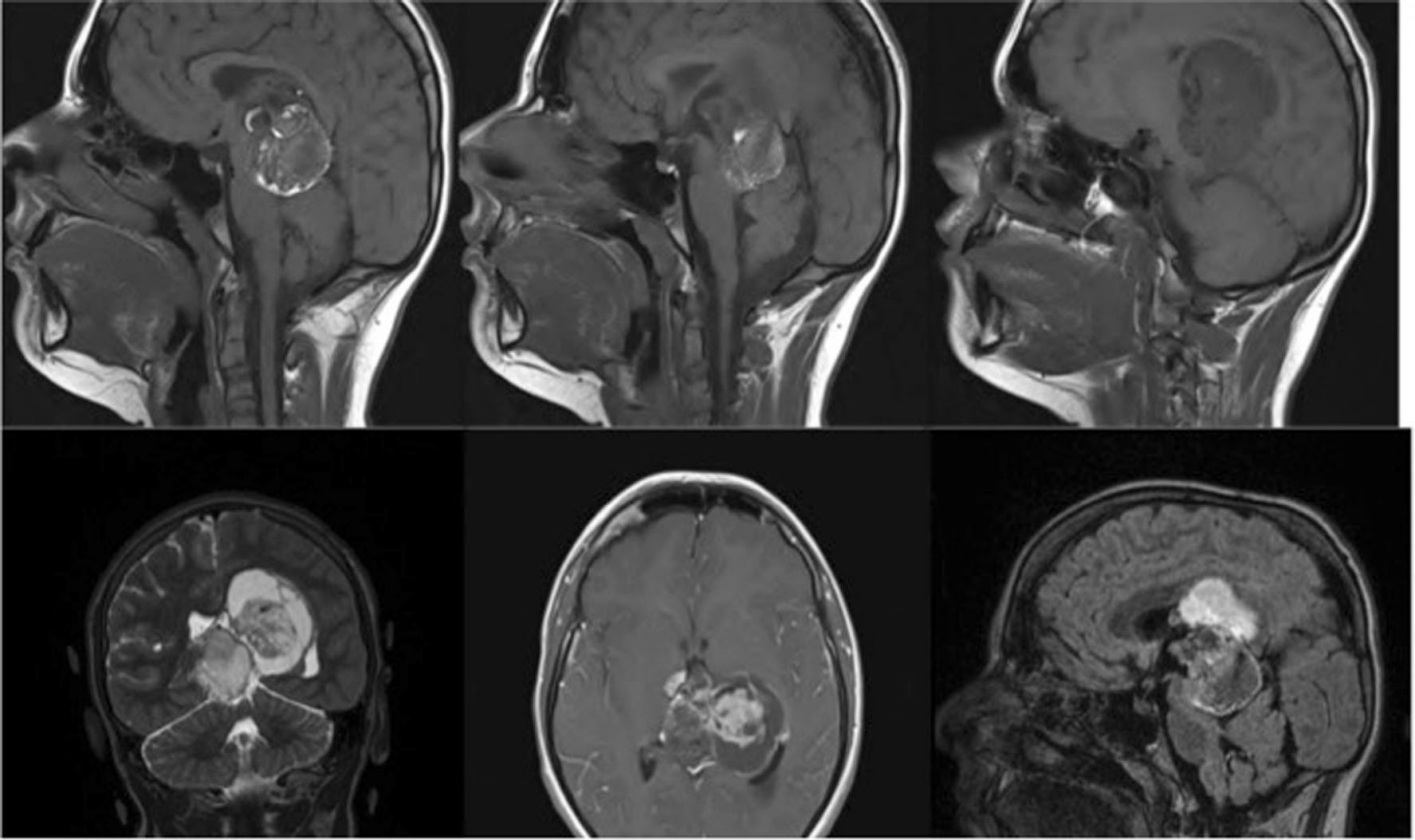
Preoperative images showing a tumour in the pineal region extending into the left lateral ventricle, compressing the tectum and causing caudal displacement of the cerebellum

A 15-year-old boy presented to our unit with Parinaud syndrome, headaches and ataxia. MRI scan demonstrated a large pineal mass extending into the lateral ventricles and compressing the tectal plate (Fig. 1). The location of the tumour would have made a single-stage complete removal by microscope very challenging. We therefore felt that an EESI approach would provide the best chance of a single-stage removal.

### Surgical technique

The patient was placed in the sitting position and head fixed in a Mayfield clamp. A routine posterior fossa approach was performed to allow a supracerebellar infratentorial approach. The craniotomy was fashioned to allow exposure of the inferior margin of the transverse sinus and allow the dura at this level to be hitched back.

Macroscopically, the arachnoid adhesions between the cerebellum and the tentorium were divided along with any small veins attaching the two structures. The cerebellum was protected by covering with large patties and then displaced inferiorly to allow introduction of the 0° endoscope and instruments. As in endoscopic skull base surgery, a “two-surgeon, four-hand” technique was used. The assisting surgeon operates the endoscope leaving the operating surgeon to use both hands for standard microneurosurgical techniques to be employed. The assisting surgeon can still use their free hand to employ an instrument to aid the surgery.

**Fig. 2:**
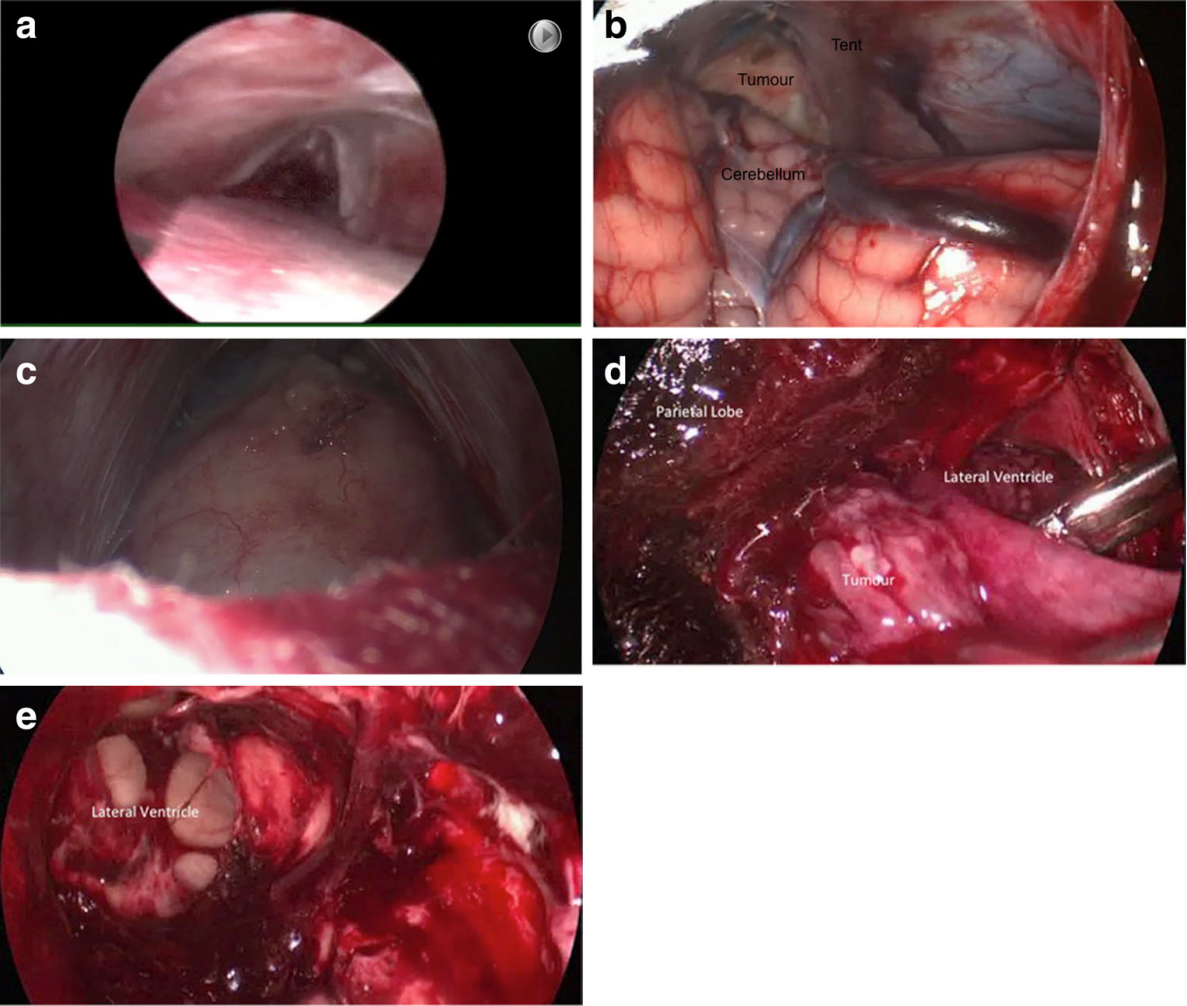
Endoscopic views of the approach and tumour. **a** Initial view with a 0° endoscope showing the tentorium superiorly, the cerebellum covered with a cottonoid inferiorly and the pineal recess anteriorly. **b** Initial view of midline aspect of tumour (cerebellum, tent and tumour labelled). **c** Closeup view of tumour with cottonoid covering cerebellum inferiorly. **d** 30° endoscope view of tumour within left lateral ventricle. **e** 30° endoscope view of left lateral ventricle after tumour excised

Figure 2a–c shows the view from a 0° endoscope of the initial approach and midline aspect of the tumour. The operating surgeon can be seen to be using instruments in both hands to allow microneurosurgical dissection. The midline component of the tumour was dissected free from the surrounding structures, debulked, and excised.

Once this aspect of the tumour was excised, the 30° endoscope was introduced and attention drawn to the lateral component (Fig. 2d). The tumour was again dissected free, gradually debulked, and excised. Figure 2e shows the cavity within the ventricle once the tumour had been excised. Histological examination confirmed a mature teratoma.

**Fig. 3:**
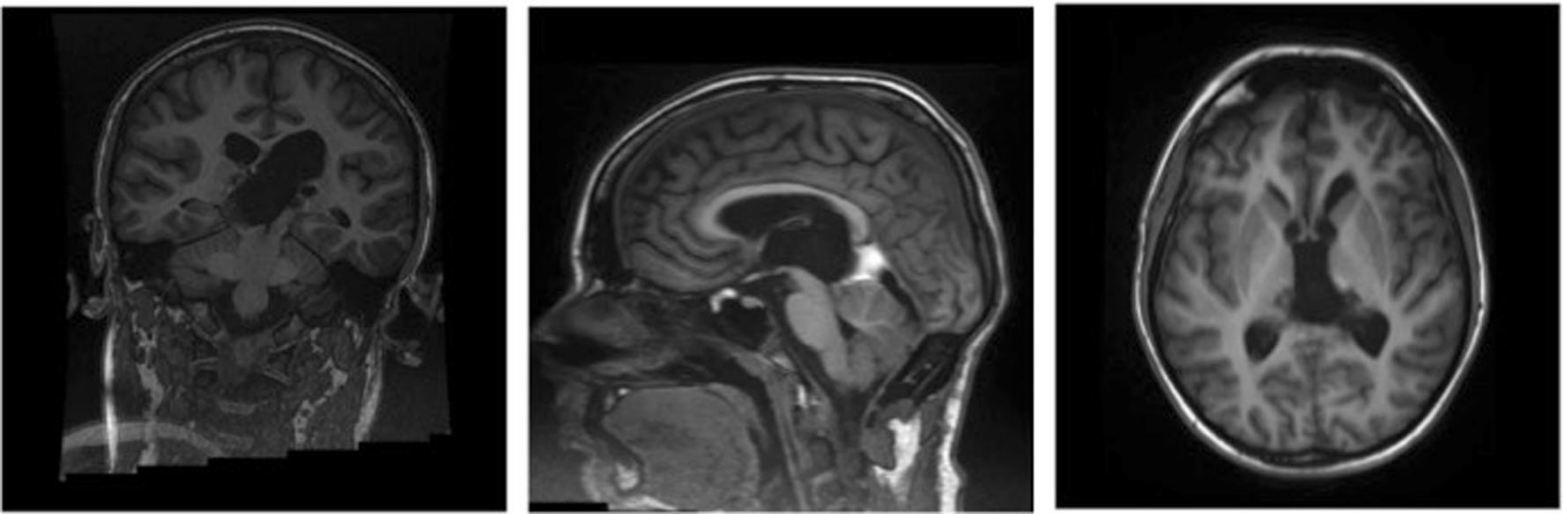
Postoperative MRI images showing complete resection of tumour at the end of procedure

After haemostasis, the dura was closed and the craniotomy secured. The wound was then closed in a standard fashion. The patient was then transferred to the MRI scanner, and an immediate postoperative scan showed complete excision of the tumour (Fig. 3).

### Progress

The patient has gone on to make an excellent recovery and remains asymptomatic. He returned back at school within 6 months. His follow-up imaging at 1 year continues to show no tumour recurrence.

## Conclusion

Endoscopic-assisted microneurosurgery is becoming increasingly commonplace. The extra advantage gained by using an extended endoscopic approach with angled endoscopes is that pineal region lesions that extend beyond the midline can be seen and accessed without the potential need for a second approach. We believe that this case highlights the benefit of using the endoscopic extended supracerebellar infratentorial (EESI) approach and it can be utilised successfully when operating on complex pineal region lesions.
